# Users’ Reactions to Announced Vaccines Against COVID-19 Before Marketing in France: Analysis of Twitter Posts

**DOI:** 10.2196/37237

**Published:** 2023-04-24

**Authors:** Alexandre Dupuy-Zini, Bissan Audeh, Christel Gérardin, Catherine Duclos, Amandine Gagneux-Brunon, Cedric Bousquet

**Affiliations:** 1 Laboratoire d'Informatique Médicale et d'Ingénierie des connaissances en e-Santé, LIMICS Sorbonne Université, Université Sorbonne Paris Nord, Institut national de la santé et de la recherche médicale, INSERM Paris France; 2 Institut Pierre Louis d'Epidémiologie et de Santé Publique Département de médecine interne Sorbonne Université Paris France; 3 Groupe sur l'Immunité des Muqueuses et Agents Pathogènes Centre International de Recherche en Infectiologie University of Lyon Saint Etienne France; 4 Vaccinologie Centre Hospitalier Universitaire de Saint-Etienne Saint Etienne France; 5 Service de santé publique et information médicale Centre Hospitalier Universitaire de Saint Etienne Saint Etienne France

**Keywords:** COVID-19 Vaccines, Social Media, Deep Learning, France, Sentiment Analysis

## Abstract

**Background:**

Within a few months, the COVID-19 pandemic had spread to many countries and had been a real challenge for health systems all around the world. This unprecedented crisis has led to a surge of online discussions about potential cures for the disease. Among them, vaccines have been at the heart of the debates and have faced lack of confidence before marketing in France.

**Objective:**

This study aims to identify and investigate the opinions of French Twitter users on the announced vaccines against COVID-19 through sentiment analysis.

**Methods:**

This study was conducted in 2 phases. First, we filtered a collection of tweets related to COVID-19 available on Twitter from February 2020 to August 2020 with a set of keywords associated with vaccine mistrust using word embeddings. Second, we performed sentiment analysis using deep learning to identify the characteristics of vaccine mistrust. The model was trained on a hand-labeled subset of 4548 tweets.

**Results:**

A set of 69 relevant keywords were identified as the semantic concept of the word “vaccin” (vaccine in French) and focused mainly on conspiracies, pharmaceutical companies, and alternative treatments. Those keywords enabled us to extract nearly 350,000 tweets in French. The sentiment analysis model achieved 0.75 accuracy. The model then predicted 16% of positive tweets, 41% of negative tweets, and 43% of neutral tweets. This allowed us to explore the semantic concepts of positive and negative tweets and to plot the trends of each sentiment. The main negative rhetoric identified from users’ tweets was that vaccines are perceived as having a political purpose and that COVID-19 is a commercial argument for the pharmaceutical companies.

**Conclusions:**

Twitter might be a useful tool to investigate the arguments for vaccine mistrust because it unveils political criticism contrasting with the usual concerns on adverse drug reactions. As the opposition rhetoric is more consistent and more widely spread than the positive rhetoric, we believe that this research provides effective tools to help health authorities better characterize the risk of vaccine mistrust.

## Introduction

### COVID-19 and Vaccine Hesitancy

Since December 2019, the COVID-19 outbreak has led governments to impose a wide range of policies to help contain the effect of the pandemic. In France, after a 55-day total lockdown from March 2020 to May 2020, restrictions were partially lifted during the summer, but they were reinstated as soon as October 2020. COVID-19 vaccines were developed since the first wave of the pandemic. Early, before their development, COVID-19 vaccines had to face the challenge of acceptance, as France is one the most “vaccine-hesitant” countries in general.

Parallel to the spread of the COVID-19 pandemic, an infodemic was observed. An infodemic is an excess of information including false or misleading information in digital and physical environments during a disease outbreak. Social media may contribute to an infodemic and to the spread of misinformation about vaccines and contribute to decision-making about vaccines. Thus, social media listening may constitute a means to predict attitudes toward a vaccine. Our hypothesis was that analyzing conversations on social media such as Twitter during the first wave of the COVID-19 pandemic could have supported the prediction of attitudes of the French population.

According to the World Health Organization (WHO), “vaccine hesitancy refers to delay in acceptance or refusal of vaccines despite availability of vaccination services” [1]. When vaccines are not available on the market, hesitancy can be substituted by mistrust. For example, “vaccine hesitancy” is relevant for the human papillomavirus (HPV) vaccine when concerns about this vaccine are reported in the literature after it is available on the market.

Questioning the relevance of vaccines has been a well-established trend in France since the end of the 19th century when the hesitancy turned political. Vaccine hesitancy today still focuses on the opposition to compulsory vaccination, opposition to government intrusion into the practice of medicine, and defense of individual liberties [2]. Arguments against vaccines are supported by different actors, and public sensitivity to these ideas has become a major political issue at both national and international levels. In France, a climate of skepticism about vaccines has been fueled by events such as the suspension of the hepatitis B vaccination on suspicion of side effects and the issue of the H1N1 vaccination campaign in which large expenditures were made but the epidemic turned out to be much less intense than expected. Another example that received major attention in the media worldwide is the association between vaccination and autism [3] supported by data that were subsequently retracted [4]. Before the vaccine rollout, 26% of the French population would refuse to be vaccinated if a vaccine against COVID-19 became available [5]. Previous attitudes of vaccine hesitancy were associated with negative opinions toward COVID-19 vaccines [6]. Another study conducted by IPSOS from October 8, 2020, to October 13, 2020 [7], revealed that, in France, 54% of respondents would get it if the vaccine was available. It was one of the worst scores among 15 countries with an average of 73%. IPSOS [7] conducted this study again at the end of 2020, from December 17 to December 20, and showed that the proportion selecting “Totally Agree” fell to 40%. Among 15 countries, France was the most refractory to the vaccine against COVID-19. This was confirmed by a study by Lazarus et al [8] that showed only 59% had positive opinions about the vaccine in June 2020.

In this paper, we explored the content on Twitter with the help of advanced machine learning techniques to identify the barriers and motivations concerning the announced vaccines against COVID-19 in France between January 2020 and August 2020. Our work aimed to assess the characteristics of users’ opinions to identify positive and negative reactions about COVID-19 vaccines and reveal main elements related to vaccine mistrust in the COVID-19 context.

In this paper, we managed to identify a set of tweets to meet the objective. The added value was as follows: (1) A selection of relevant keywords for the field of anti-COVID vaccines is proposed, which combines the computation of embeddings with FastText and their representation by means of a principal component analysis, and (2) topics are identified in the tweets that could help explain mistrust of vaccines and counter them with negative argument.

### Using Social Media to Monitor Vaccine Resistance

The period of the COVID-19 crisis favored the publication of numerous surveys of European citizens to assess intentions to vaccinate against COVID-19 and to identify the categories of individuals most susceptible to vaccine resistance [9]. These studies were based on controlled statistical methods in which respondents are constrained by a limited number of answers predefined by the investigators [10]. As a result, reasons for vaccine hesitancy other than those proposed in the controlled studies cannot be detected. However, the public is exposed to new events on a daily basis, and additional reasons for vaccine mistrust may emerge rapidly and not be captured by static resources.

Real-time monitoring of social media can be an indicator of society’s hottest emerging issues. As the exhaustive analysis of a large volume of messages is impossible, the use of recent advances in natural language processing (NLP) has become the actual trend. Sentiment and opinion analysis regarding the COVID-19 pandemic benefited from recent automatic approaches to social media analysis. For example, topic modeling was used on Twitter by Wang et al [11] to analyze public opinion toward COVID-19 in California and New York and by Luo et al [12] who performed a similar analysis on HPV vaccination. Sha et al [13] used dynamic topic modeling to track governmental decision-making regarding risk, testing, and treatment based on tweets by US governors and presidential cabinet members. Several infodemiology studies applied machine learning approaches to analyze social media. Daughton et al [14] used supervised learning classifiers to identify human behaviors relevant to COVID-19. Similarly, Chen et al [15] used dimension reduction and cluster analysis to support comparison between viral COVID-19 posts on Twitter and Sina Weibo, a non-English–speaking platform in China. In some cases, multiple artificial intelligence approaches can be used to construct an observation framework, such as in the study by Adikari et al [16] in which a combination of several machine learning approaches, including NLP, word embeddings, and Markov models, was proposed to investigate COVID-19–related emotions.

Exploring vaccine hesitancy through online posts on social media is inspiring. Although some studies focused on qualitative analysis of a limited number of posts [17,18], others employed semiautomatic approaches such as in the study by Massey et al [19], which used content and network analysis to study misinformation about the HPV vaccine. Recently, the use of automatic approaches based on NLP has become frequent for quantitative studies about vaccine hesitancy. For example, for sentiment analysis about the HPV vaccine, Skeppstedt et al [20] used topic modeling on discussion forums, and Zhang et al [21] used transfer learning on Twitter posts. Similar approaches were applied to analyze vaccine hesitancy in the COVID-19 context. Hussain et al [22] used deep learning and NLP to analyze public sentiment toward COVID-19 vaccines based on a set of Twitter and Facebook posts from the United Kingdom and the United States. Kwok et al [23] used topic models based on latent Dirichlet allocation [24] for sentiment analysis about the COVID-19 vaccine among Australian Twitter users. Such studies are specific to the cultural and political context that affected decision-making in vaccination policy.

### Using Pretrained NLP Models to Support Social Media Monitoring

To our knowledge, most social media studies regarding COVID-19 vaccine hesitancy in France are qualitative and do not benefit from advanced and efficient machine learning.

There are now many pretrained NLP models available, depending on the languages or texts used during training. Models in English were created to facilitate research, such as COVID-Twitter-BERT [25], which is trained on English tweets mentioning the COVID-19 pandemic. Arnaud et al [26] described previous work on the French adaptation of Bidirectional Encoder Representations from Transformers (BERT): CamemBERT and FlauBERT. The objective was to perform unsupervised learning, while we focused on supervised learning. Blanc et al [27] compared the performances of CamemBERT and FlauBERT to build a chatbot, and Sauvayre et al [28] used CamemBERT to classify the opinion of Twitter users on COVID-19 vaccines. The BERT language model is therefore usable for the French language and could be adapted for the classification of tweets.

### Mistrust About COVID-19 Vaccines and the Opportunity With Social Media Listening

Mistrust about COVID-19 vaccines has spread widely across social media. Consequently, its influence was able to reach a large part of the population. This mistrust situation was causing concern for health authorities, including the WHO, which listed vaccine mistrust as one of the 10 biggest threats for global health in 2019 next to the threat of a pandemic [29].

According to Taylor et al [30], there are many reasons for this mistrust: One may be doubtful of the vaccine benefit; there may be concerns about long-term, unexpected side effects; marketing of vaccines may be considered as a mere commercial operation in which vendors are profiteering from patients; and one may prefer natural immunity rather than getting immunity from the vaccine. Other studies have considered conspiracy theories as an element influencing the decision to get vaccinated [31]. Examples of these theories in the context of COVID-19 are Bill Gates’ intention to create a “global surveillance state” [32] and the economic motivation of the “Big Pharma” vaccine industry [33].

Analyzing social media can facilitate the evaluation of adherence to a potential COVID-19 vaccine. As we mentioned in the Introduction section, part of the population has doubts about the nonauthorization of chloroquine for the treatment of COVID-19. These doubts could have been supported by the highly mediatized “Lancet Gate” in which the WHO urgently stopped trials on chloroquine and hydroxychloroquine as a treatment against COVID-19 based on scientific papers that used corrupted data and were retracted shortly later [34,35].

The high accessibility to social media today highlights that popular news on this resource can reach an important number of people in a short time [36]. The general director of the WHO declared in 2020 that the WHO must deal with the infodemic in addition to the pandemic [37]. A new report published by the Centre for Countering Digital Hate noted that 31 million people follow antivaccine groups on Facebook, with 17 million people subscribing to similar accounts on YouTube [38]. Such accessibility to a large volume of information can help identify public views. However, this resource cannot replace more controlled survey methods because of the inherent selection bias on one hand and the uncontrolled spread of false information via this resource on the other hand [39].

## Methods

### Overview

**Figure 1:**
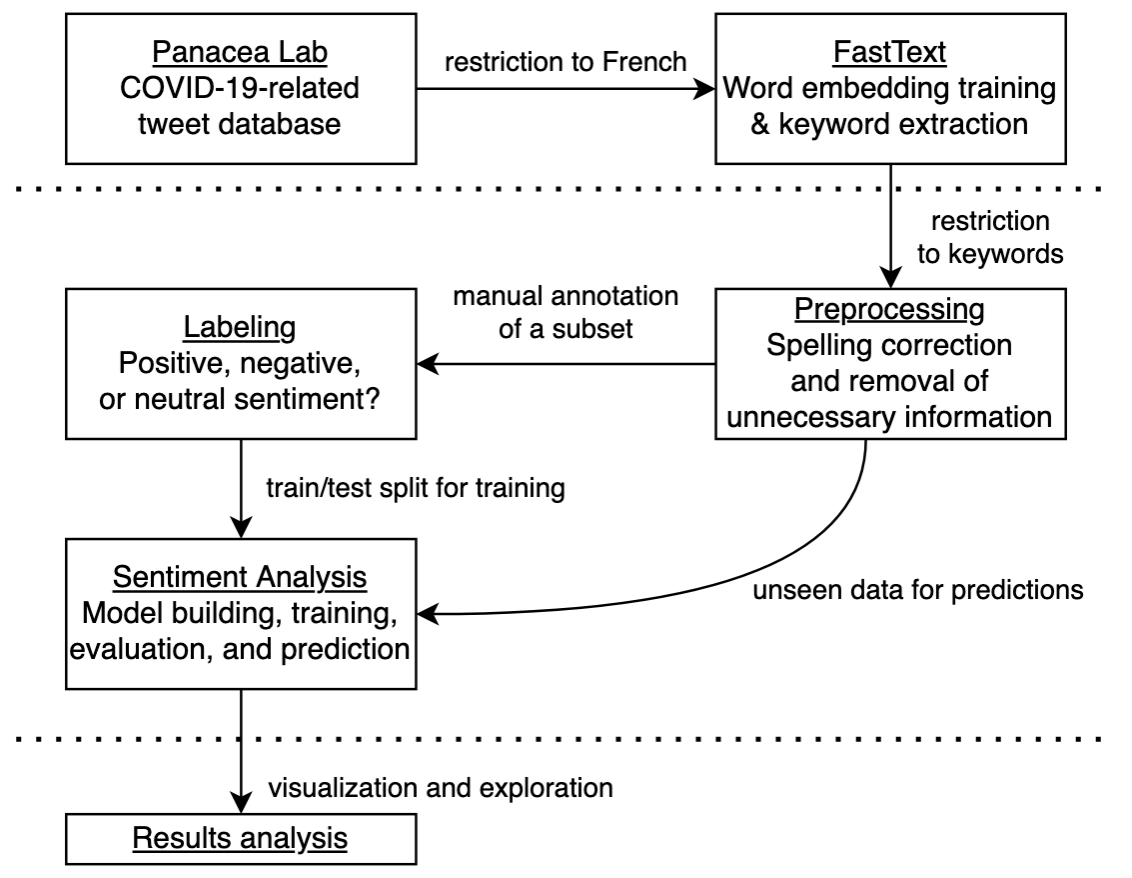
Summary of the study methodology, with the different phases separated by horizontal lines.

This study was conducted in 2 steps that were designed to be accessible and easily adapted to any other subject at a specific time. The first step consisted of constructing a data set of French tweets related to COVID-19. From this data set, a subset of keywords relative to the word “vaccin” (vaccine in French) was selected by exploring embeddings with a distance close to this word. By restricting the data set to the tweets containing at least one of these keywords, a data set related to the topic of vaccine in French during the COVID-19 pandemic was reached. The second step focused on sentiment analysis. As this part required machine learning, a small part of the restricted data set needed to be hand labeled to fine tune a pretrained model. After training and evaluating the model, label prediction was performed on the whole restricted data set, and the predictions were explored in terms of vocabulary and timeline. These steps are summarized in Figure 1 and explained in detail in the subsections hereafter.

### Data Collection

Data were collected before vaccines were available on the market. About 700 million unique tweets were identified by the Panacea Lab team at Georgia State University [40] for the period between February 2020 and August 2020 using a selection of keywords that were mainly COVID-19 designation variants, and the corresponding IDs were made available on GitHub. A new version is made available every week and collects approximately 3 million tweets per day. Once restricted to tweets in French, the database has yet to be restricted to the vaccination topic. We did not use Twitter's localization function to verify that the tweets were written in France because the information was often missing.

The data from the Panacea Lab have the advantage of having been collected constantly since the start of the pandemic using inclusion criteria that can be reproduced over time. Panacea Lab provided a public set of tweet IDs of COVID-19–related tweets. The Twitter application programming interface (API) was needed to get twitter contents based on these IDs. The advantage of using a public, prefiltered data set is the possibility to eventually compare the results to other studies using the same data set.

### Data Refinement Based on Word Embeddings

Word embeddings are a way of representing a word in the vocabulary in a mathematical space. Words are transformed into vectors of a fixed number of dimensions to embed information about their meaning in the corpus. Thus, words that are close in meaning will be close in distance in this space. The choice of the word embeddings model is based on the properties and specificities of the data, as they influence the performance of the algorithms using them.

For this purpose, a word embeddings model was trained on the whole French tweet data set to catch emerging words that would not exist in a training data set of pretrained algorithms or pretrained word embedding algorithms.

### Data Set Generation Using a Fine-Tuned FastText Model

Since the data are composed of short, noisy messages with uncertain spelling, the word embeddings generated by FastText offer significant advantages. Notably, this method is considered to be fast, enables words with a similar spelling to be brought together by using parameter sharing, and can provide better performance when the vocabulary contains many syntactic or orthographic variations of the same word. Most word embedding models learn a vectorial representation from the word’s context, but as FastText also learns additional embeddings at the character level of the word, the decomposition of an unknown word enables it to learn more relevant embeddings for rare words. The aforementioned technique, which is named parameter sharing, is an advantage since most models do not address the diversity of morphologically rich languages such as French. Accurate word representations are difficult to learn since many word forms occur too rarely in the training corpus. Parameter sharing also enables the handling of uncertain spelling observed in tweets.

As word embedding models come with hyperparameters, tuning FastText is necessary to fit the corpus at best. The metric used for that task is a criterion named the Discounted Cumulative Gain (DCG), which considers a user-defined collection of word pairs that are known to be close in meaning or context and computes the score of the cumulative closeness of the embeddings associated with the pairs. One example was the pair of French words “covid” and “coronavirus.” The list of 24 word pairs in French we used and their translation in English are available in Multimedia Appendix 1. The higher the DCG, the more the model is expected to fit the corpus.

To complete the restriction of the French tweets to a vaccine-centered data set, a semantic field of the French word “vaccin” was built: By iterating from the word “vaccin,” words close in the embedding space were added to the semantic field by computing their distance. This set of keywords is therefore composed of the closest words in distance to the word “vaccin” in the embedded space, truncated with a user-defined threshold. First, we selected a couple of words close to “vaccin.” Then, we used the group of words composed of “vaccine” and its close neighbors to find words that are close to this group. Finally, we iterated by making the group grow by integrating more neighbors until there were no neighbors considering the user-defined threshold. The final restricted data set was then composed of tweets containing at least one of those keywords. These keywords were also used to explore the topics surrounding the vaccination topic using principal component analysis (PCA).

### Data Preprocessing

Data preprocessing was designed to be as minimal as possible and focused on 2 main tasks. The first was to delete unnecessary information, and the second was to lower the noise in texts.

First, the following steps were applied to the restricted data set: URLs, punctuation, and special characters were removed. Tweets were then split into lists of lowercased words, and words that did not add any real value to the meaning (stop words) were also removed.

In addition, some words such as “hydroxychloroquine” have many spelling variations. In order to suppress part of the noise to obtain better performance, a spelling correction step was applied. It consisted of comparing each word to a custom French dictionary and then correcting the 3000 most common mistakes; it could correct approximately 70% of the misspelled words in the corpus. Thus, a dictionary of 3482 words was manually created to correct this noise.

Finally, 1 Twitter user appeared to likely be a bot by repeatedly posting 3 identical texts mentioning different users each time. It represented a total of 2343 tweets published in a very short period. This user was therefore deleted in order not to bias the results of the study.

### Model Building and Training

After the preprocessing steps, an extended analysis to explore users’ sentiments about vaccination was initiated using advanced machine learning models. Some pretrained models in French can be adapted to a supervised classification task. We chose transfer learning, which allows learning with a limited amount of data or low computational capacity. Thus, as the model already has a sufficient understanding of the French language, it is not necessary to attain a large quantity of hand-labeled tweets.

Most recent language models are based on the BERT model [41]. As resources trained on tweets mentioning the COVID-19 pandemic are not available in French, it was necessary to fine tune existing models. Several pretrained models are available, with different sizes of architecture that are specific to one or more languages or that are trained on a particular type of text. To use these models for sentiment analysis, we chose to construct a classifier at the output of a BERT-type model to determine which of the classes was the most likely for each tweet. The BERT-type model chosen here was CamemBERT [42], to which a linear classification layer was added. This linear layer learns the best multidimensional linear regression to perform on the output of the BERT-type model to obtain the desired predictions. Many other classification layers can be evaluated to improve performance. Moreover, this implies that the training is supervised.

This study classified tweets into 3 categories: positive, negative, or neutral sentiment. Tweets labeled as positive mentioned the announced vaccine in an optimistic, confident way and often diffused encouraging news on the subject. The negative tweets evoked the potential vaccine in a mistrustful and possibly conspiratorial way, and they sought to warn of its possible risks or manipulation or to relay information spreading doubt about its effectiveness. The neutral ones were unrelated to vaccines or did not contain any judgment about them.

For training, a total of 4548 tweets (4548/344,000, 1.3% of the restricted data set) were labeled by a single annotator, of which 26.9% (1223/4548) were negative, 21.2% (965/4548) were positive, and 51.9% (2360/4548) were neutral. The classification task was therefore imbalanced, and this had to be addressed by using specific methods. First, a stratified training method was required so that the training and test sets respected the proportion of each class. In order to compare methods, metrics must ensure that each class is best predicted not only as a whole but also class by class. Precision (ratio of predicted items that truly belong to this class) and recall (ratio of correctly predicted items among the known items of this class) were used to measure the performance of our method class by class, in addition to the *F*_1_-score, which is their harmonic mean. Accuracy (ratio of correct predictions) is the metric used to give a general appreciation of the performance of the models. As a matter of performance comparison, a common sentiment classification model using a combination of term frequency-inverse document frequency (TF-IDF) and multinomial naïve Bayes was used.

### Implementation

We used the following Python packages: sklearn, fastText, pytorch, pandas, kedro, and transformers. We used SSH access and Jupyter notebooks on a server consisting of a 28 core/56 thread microprocessor (Intel x64), 256 GB of memory, 1 TB of NVMe (nonvolatile memory express) storage, and 2 Nvidia GPUs of 2432 cuda core each (GeForce GTX 1070 Ti) with a Linux Debian 9.9 operating system (see the Acknowledgments section).

### Ethics Approval

This study received approval from the ethics committee at the centre hospitalier universitaire de Saint-Etienne under the Institutional review board number 1412020/CHUSTE.

## Results

### Data Collection Based on Word Embeddings

The initial data set restricted to French tweets consisted of 894,315 unique words for 4,020,525 tweets. After training word embeddings for 300 dimensions, relations between words were explored, and 69 keywords were identified as the semantic concept of the word “vaccin.” The list of the 69 keywords and their translation in English are available in Multimedia Appendix 2.

**Figure 2:**
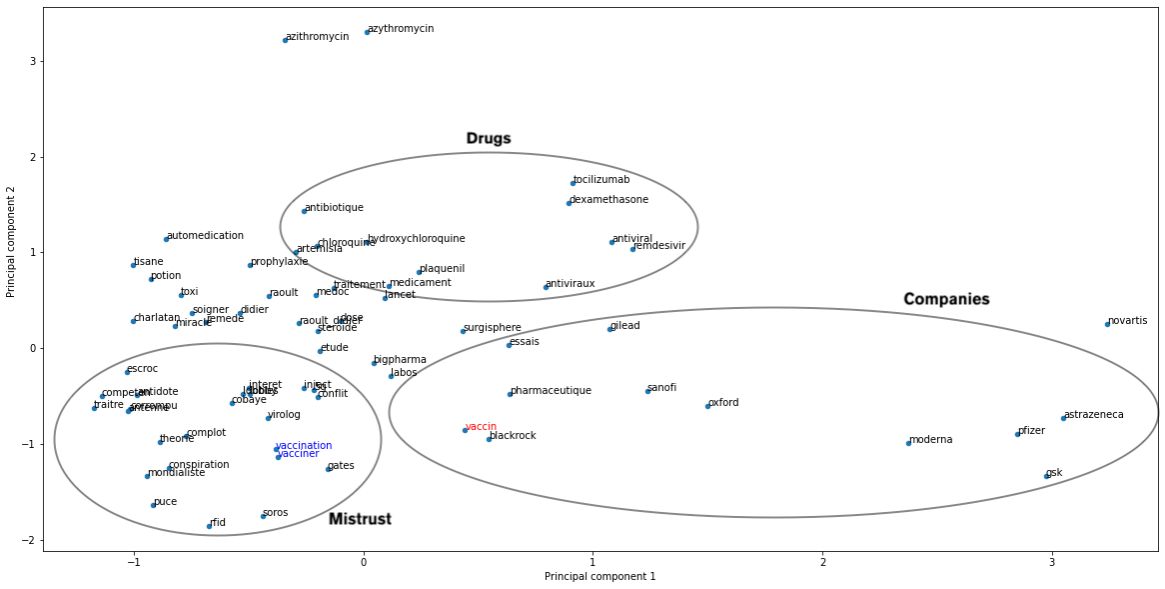
2D projection of the 69 identified keywords, with the starting word “vaccin” in red and “vaccination” and “vacciner” in blue for readability reasons, 3 hand-drawn ellipses highlighting sets of words present in the same area of the figure and showing that elements of a similar nature (eg, mistrust, drugs, or companies) can be grouped together. The addition of ellipses reflects only the interpretation of the authors and is not based on any mathematical theory.

Using PCA only as a visualization tool, we displayed a projection of the vector representations of each keyword on a shared 2D plan (Figure 2). This projection seems to group keywords into 3 groups surrounding the word “vaccin,” which could be summarized as potential treatments (top left; eg, antibiotique, antiviraux, artemisia, azithromycin, chloroquine, dexamethasone, hydroxychloroquine, plaquenil, remdesevir, and tocilizumab), conspiracy (bottom left), and pharmaceutical companies (right; eg, AstraZeneca, Gilead, GSK, Moderna, Novartis, Oxford, Pfizer, and Sanofi). The projection of the terms “vaccination” and “vacciner” (to vaccinate) sets these words among the words related to conspiracy theory terms like “charlatan” (charlatan), “cobaye” (guinea pig), “complot” (conspiracy), “conflit” (conflict), “conspiration” (conspiracy), “corrompu” (corrupt), “escroc” (crook), “intérêt” (interest), “lobby” (lobby), “mondialiste” (globalist), “puce” (chip), “théorie” (theory), and “traitre” (traitor), as well as mentions of Bill “Gates” and George “Soros.” Although “vaccination” and “vacciner” have the same root as “vaccin,” the embeddings captured nuances in their contexts. This information already unveils some polarity that is associated with the vocabulary.

Tweet filtering based on word embeddings provided a specific data set by restricting the collection to 344,000 tweets in French (around 9% of the initial French data set). Filtering based on the “vaccin” word alone would have generated a subset of 75,000 tweets, thus ignoring a large number of tweets potentially related to aspects of vaccine mistrust. The results presented in the rest of this paper concern the 344,000 French tweets related to a potential vaccine against COVID-19.

### Sentiment Analysis Model Evaluation

**Figure 3:**
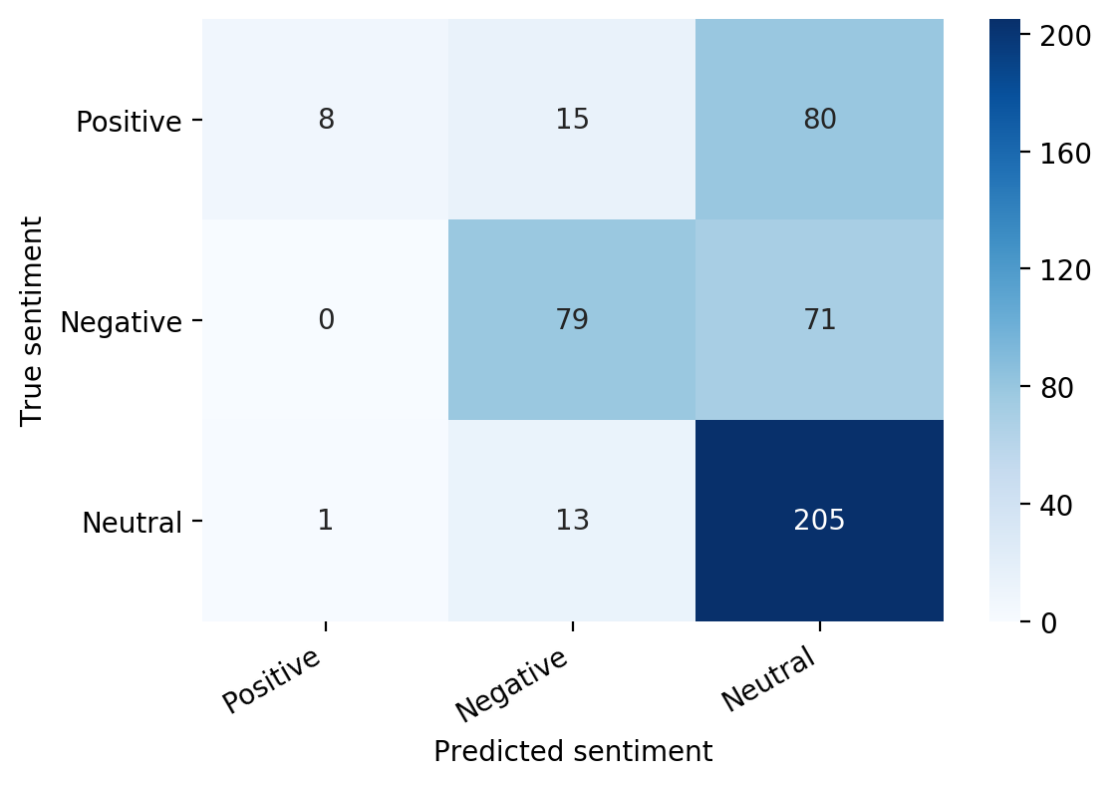
Confusion matrix for term frequency-inverse document frequency (TF-IDF) and multinomial naïve Bayes.

To be able to compare the performance of a more complex model using CamemBERT and a linear layer classifier, it is necessary to evaluate very simple models and to know their performances. We chose 2 simple baseline models. Since the classes are very imbalanced in favor of the neutral class, the simplest model is to predict only this neutral class. This first baseline model had an accuracy of 0.52, and any other model must achieve better performances than this. The second baseline model was more meaningful while still being very simple. It combines a document embedding vectorization to TF-IDF features and a classifier named multinomial naïve Bayes. A document embedding is a vector representation of each document, according to the words of which it is composed. It is then used by the classifier to predict 1 of the 3 classes. After training, the metrics of this baseline are summarized in the following table (Table 1). Although the precision of the model in predicting a positive or a negative tweet was 0.89 and 0.74 respectively, the recall fell to 0.08 for the positive tweets and 0.53 for the negative tweets. Moreover, the prediction of the neutral class had a high recall of 0.94 but a low precision of 0.58 compared with the precision of positive and negative tweet predictions. Positive and negative tweets seemed to be misclassified into the neutral class, which therefore had a weaker precision. The confusion matrix confirmed those conclusions (Figure 3).

**Table 1:** Classification summary for term frequency-inverse document frequency (TF-IDF) and multinomial naïve Bayes (MNB), as well as CamemBERT with the linear layer.

Classification	Precision			Recall			*F*_1_-score		
	MNB	CamemBERT	MNB		CamemBERT	MNB		CamemBERT	
Positive	0.89	0.64	0.08		0.73	0.14		0.68	
Negative	0.74	0.75	0.53		0.77	0.61		0.76	
Neutral	0.58	0.83	0.94		0.75	0.71		0.78	
Macro-average	0.73	0.74	0.51		0.75	0.49		0.74	
Micro-average	0.70	0.76	0.62		0.75	0.56		0.75	

**Figure 4:**
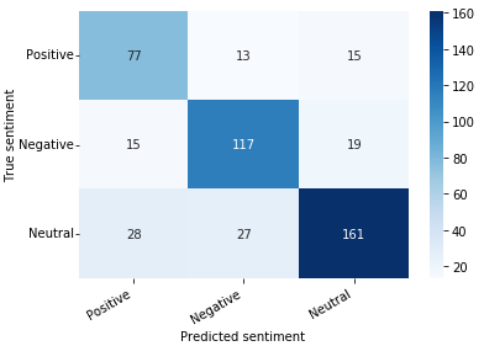
Confusion matrix for CamemBERT and a linear layer.

The performance of the sentiment analysis model proposed in this paper, which combines CamemBERT and a linear layer, is also summarized in Table 1. All *F*_1_-scores are higher than those of the baseline. The proposed model therefore obtained fewer misclassifications for each class than the baseline. This conclusion is particularly visible for positive tweet predictions, for which there were fewer missed true positives (recall). Those results were also confirmed by the confusion matrix (Figure 4). However, the margin of error was still rather high, as the accuracy was only 0.75 compared with 0.62 for the multinomial naïve Bayes model. However, it is important to keep in mind the ambiguity of most tweets, whose annotation is subject to personal interpretation.

### Characteristics of Vaccine Mistrust

**Figure 5:**
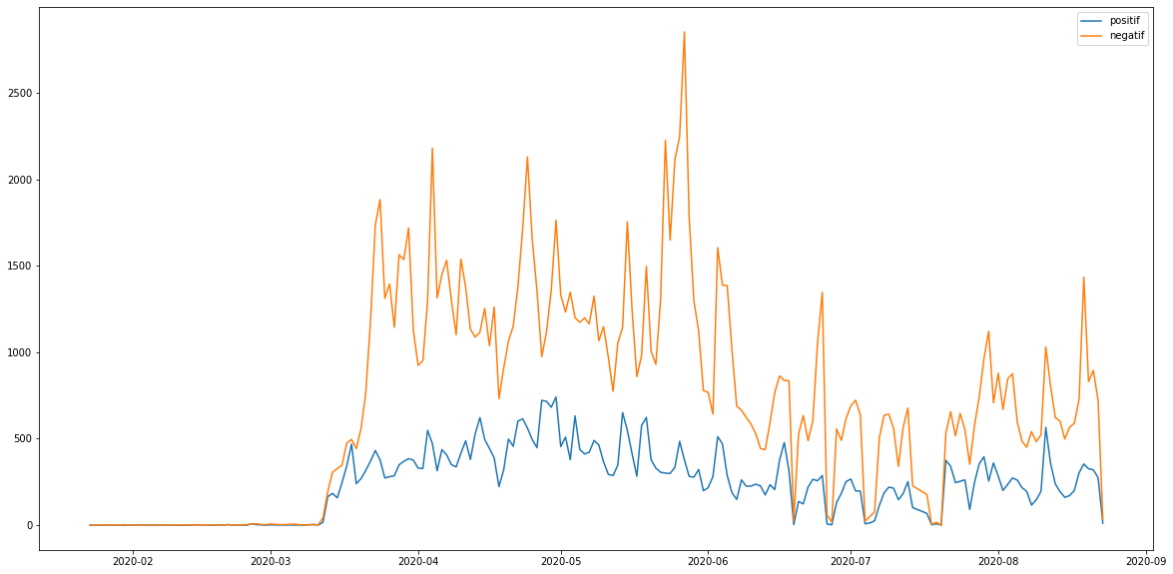
Counts of positive (blue) and negative (orange) tweets per day during the study period.

In order to summarize the information obtained from this classification, 2 representations were chosen: the temporal presentation of the tweet counts per class and the presentation of the most common words for each sentiment. As neutral tweets provide little information on the reasons for vaccine mistrust, the visualizations will only focus on the positive and negative tweets. Figure 5 displays the counts of positive and negative tweets per day during the study period. Word embeddings using FastText allowed us to identify a broader range of arguments for vaccine mistrust. However, this method included more negative opinion than positive opinion, compared with the counts per day if only tweets containing the word “vaccin” were considered. The wider the vocabulary is, the weaker the signals of vaccine mistrust that are detected.

It is thus possible to distinguish days with a higher activity than usual for both sentiments. We assumed that evaluation should focus on days when counts of tweets showing positive or negative sentiment showed a polarization of the users’ opinions, rather than days when the numbers of tweets were high but mostly neutral. As trends sometimes fluctuate a lot from one day to the next, there seemed to be temporary events of which users quickly take advantage.

**Figure 6:**
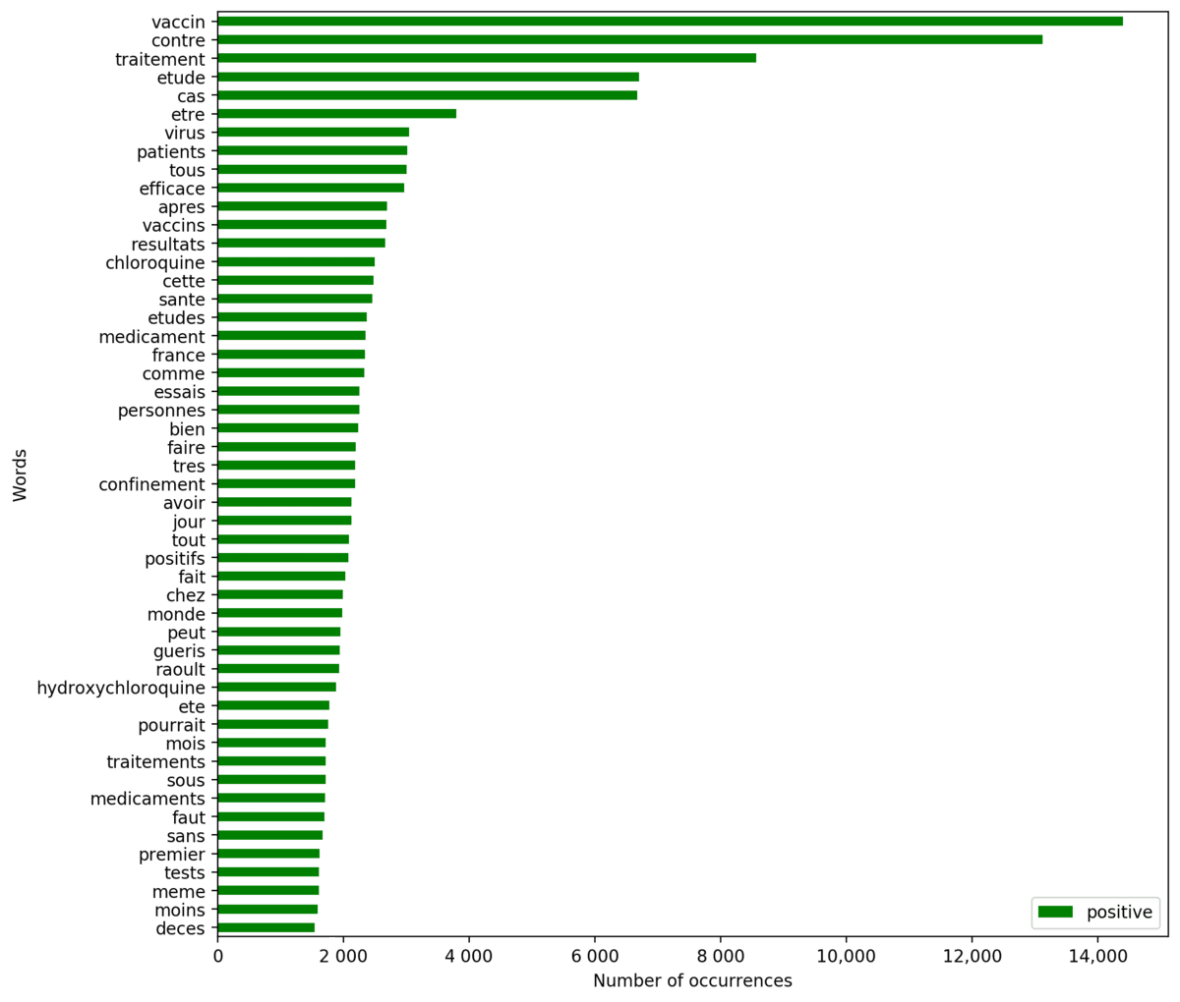
Most frequent words for positive tweets.

**Figure 7:**
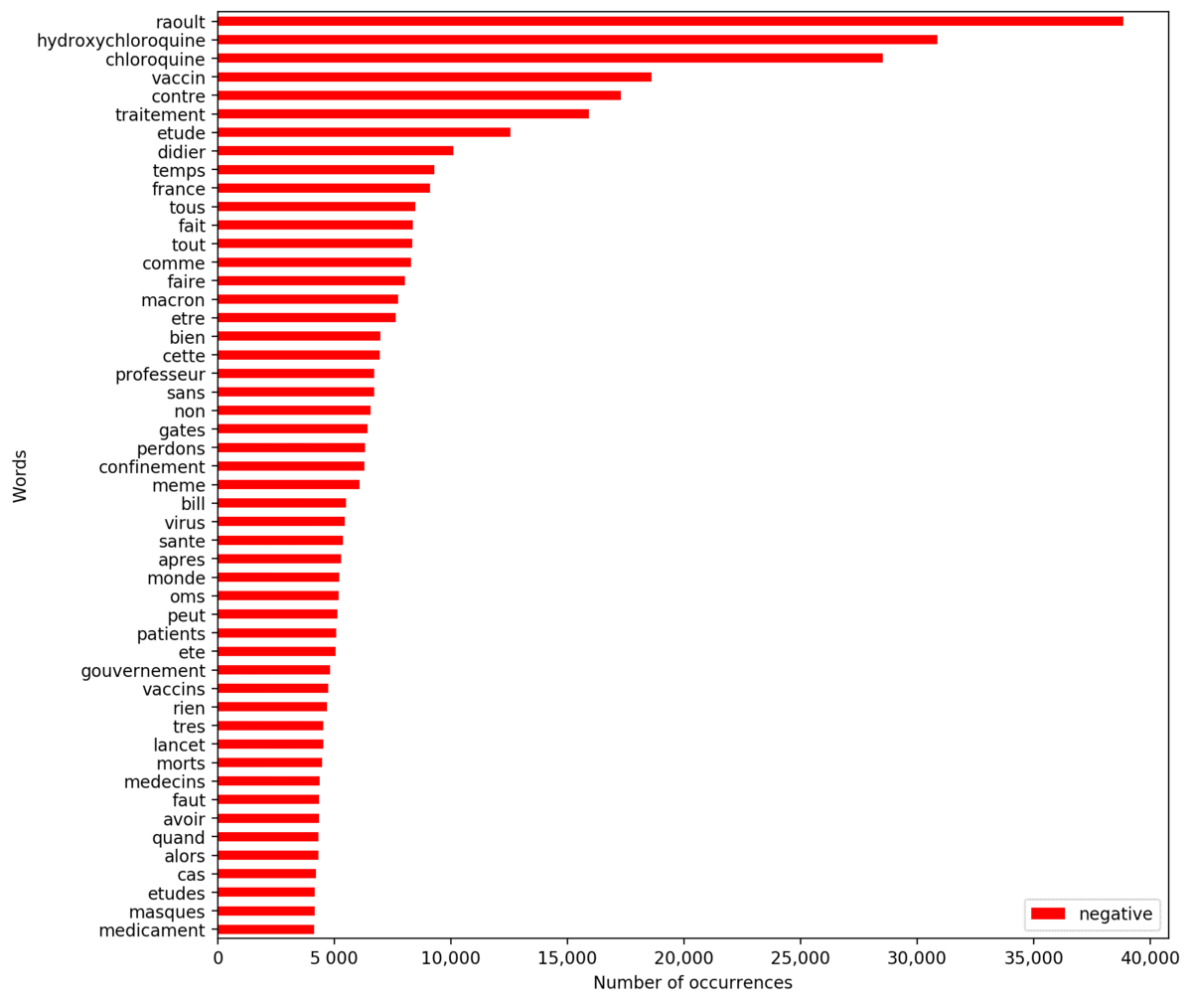
Most frequent words for negative tweets.

**Figure 8:**
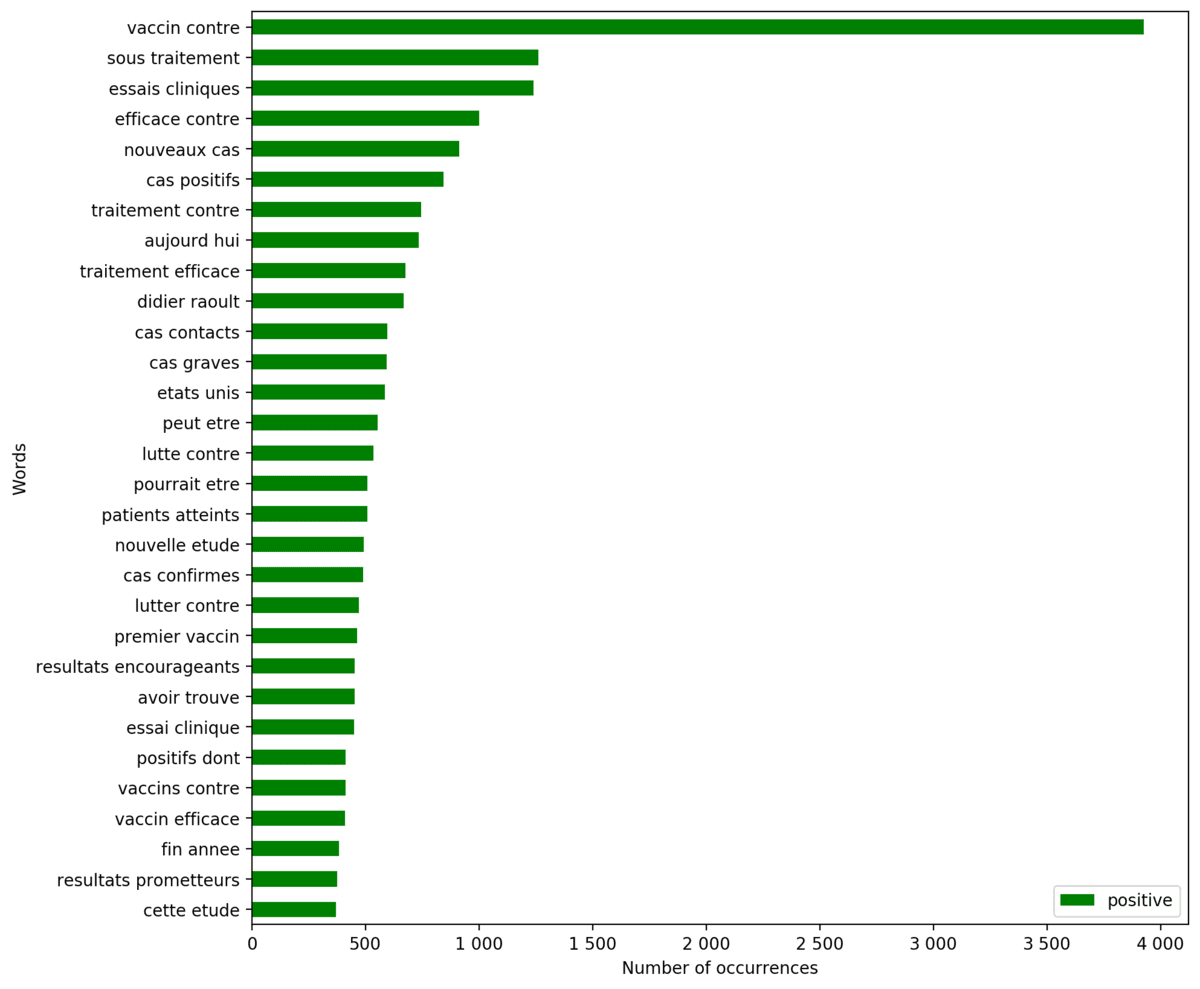
Most frequent bigrams for positive tweets.

**Figure 9:**
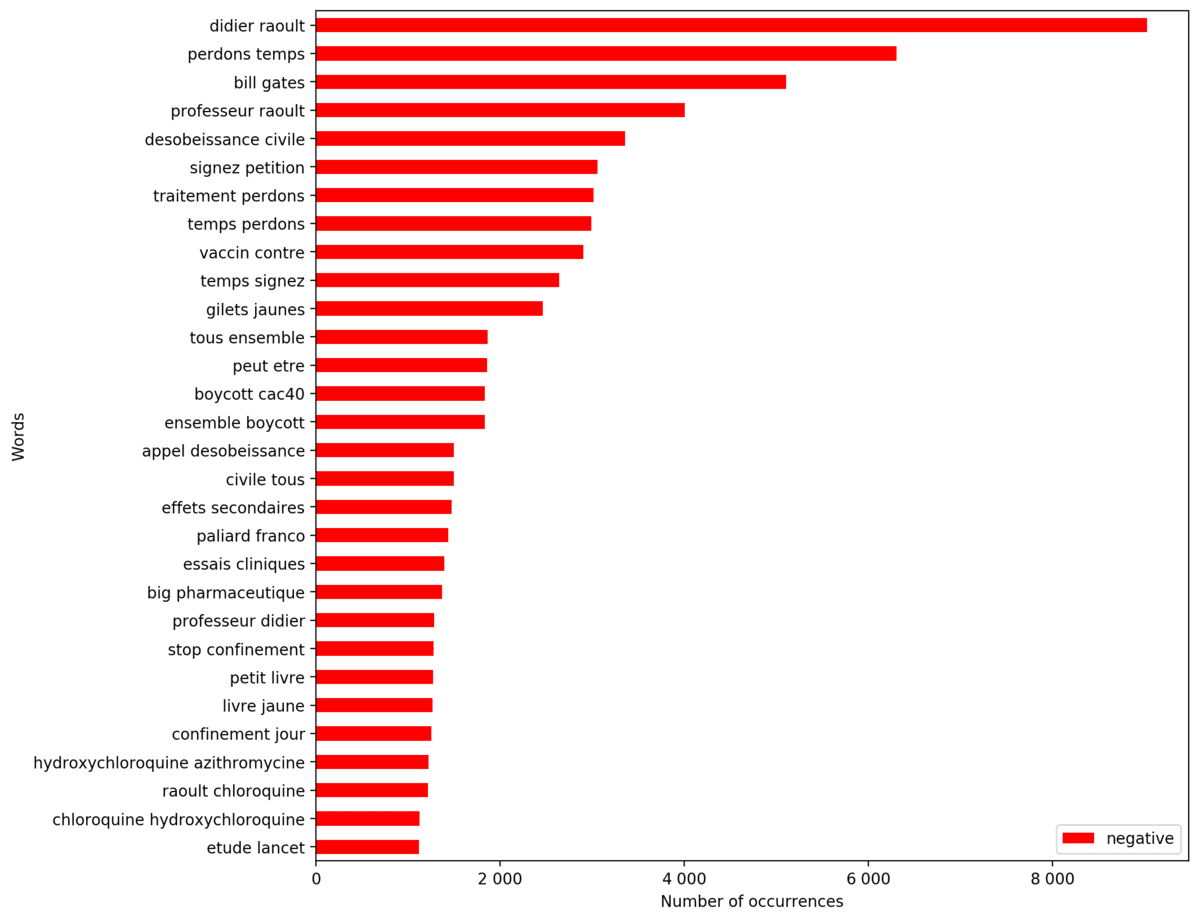
Most frequent bigrams for negative tweets.

Simultaneously, it is possible to explore the most used vocabulary per sentiment. The following figures present the words (positive tweets in Figure 6 and negative tweets in Figure 7) and bigrams (positive tweets in Figure 8 and negative tweets in Figure 9) that were most frequent per sentiment. Discourses were more homogenous in the negative tweets, where they focused on alternative treatments (eg, “hydroxychloroquine”), political contestation of the government (eg, “désobéissance civile” [civil disobedience], “gilets jaunes” [yellow vests, named after the yellow high-visibility vests worn by protestors during a movement that emerged in France in October 2018]), and conspiracies (eg, “bill gates,” “boycott cac40”). “Raoult,” “chloroquine,” and “hydroxychloroquine” were the monograms the most discussed in negative tweets. “Didier Raoult” and “Professor Raoult” were the first and fourth bigrams, respectively, that were the most discussed in negative tweets.

Meanwhile, discussions about research advances were among the most common in the positive tweets. The most frequent positive terms focused on the efficiency or early results of the candidate vaccines (eg, “efficace contre” [effective against], “résultats encourageants” [encouraging results]). Nevertheless, positive tweets also communicated some terms like “chloroquine” that were mostly attributed to negative tweets.

## Discussion

### Main Findings

The objective of this study was to characterize the opinion of Twitter users on the subject of a potential vaccine against COVID-19. After a review of the literature in both the medical and machine learning domains, the chosen steps were to extract a set of relevant tweets, then to exploit them from a sentiment analysis perspective, and finally to explore these results. Using a vectorial vocabulary representation method proved to be a powerful way of broadening the lexical concept of vaccination during this pandemic. First, we implemented word embeddings using FastText and could identify 69 keywords representative of issues relative to vaccination against COVID-19. The relevance of these keywords was confirmed by their relative position on a PCA and their relation with news about COVID-19 in France (eg, a study questioning the use of chloroquine published then withdrawn from the Lancet or conspiratorial theses such as the participation of Bill Gates and 5G chips in vaccination campaigns with the aim of taking control of people).

Second, we implemented a classifier based on CamemBERT, a French adaptation of BERT, a language model part of transformers with an *F*_1_-score of 0.75 for predicting the opinion of Twitter users about potential vaccines against COVID-19. This allowed us to more precisely characterize tweets that presented positive or negative opinion on vaccination. We found that tweets with a negative opinion presented more polarized and political arguments than tweets with a positive opinion, and the role of treatments other than vaccines such as chloroquine was a major issue when discussing mistrust of vaccines.

### Related Work

Most papers that evaluated tweets for extracting insights about vaccination against COVID-19 used an unsupervised approach, mainly topic models with latent Dirichlet allocation [24], which means that a training set was not necessary. When sentiment analysis was performed, authors used a pretrained classifier most of the time, especially relying on Valence Aware Dictionary for Sentiment Reasoning (VADER) [43], which is available in the NLTK Python library. Using a pretrained classifier expedites classification but presents the inconvenience that it is not specialized for the study domain. Transformers are currently acknowledged as presenting the best performances in several NLP tasks. Tweets are more difficult to classify due to their characteristics: They are limited to 280 characters but can be much shorter; they are not necessarily written with correct syntax; and they can contain abbreviations, misspellings, and slang words [44]. We also observed that the results of predictions are generally significantly better for correctly written texts such as PubMed abstracts, than with tweets, for example [45].

In previous work, several custom algorithms were proposed, such as (1) deep learning with a CamemBERT model [28], BERT [46,47], RoBERTa [48], FastText [49], convolutional neural network–long short-term memory with word2vec embeddings [50] or (2) machine learning with naïve Bayes [51] or decision tree [52] models. Off-the-shelf sentiment analysis models include Amazon Web Services Comprehend sentiment analysis [53] and VADER [54-60], which is a Python lexicon and rule-based sentiment analysis tool [43]. In a recent evaluation of 11 sentiment analysis tools on 7 social media data sets, He et al [61] observed that these tools do not provide results that are accurate enough to aid in public health decision-making. Among all tools, VADER was one of the 3 top performers.

Our *F*1-score was 0.75, but it is at the same level as the best scores obtained in other papers in which this score is available. Some papers provided only partial results that did not allow a comparison, and most provided no metrics for the classifier. Li et al [55] obtained *F*_1_-scores of 0.76 for “unrelated” (class 0), 0.50 for “positive” (class 1), and 0.37 for “negative” (class –1), which means that we obtained better results if we consider the average of the scores of the 3 classes. Mønsted and Lehmann [49] obtained a micro-averaged *F*_1_-score of 0.762 for a 3-class prediction, which is a good result considering that the authors used Amazon’s Mechanical Turk platform. Sauvayre et al [28] classified tweets according to the opinion for or against users and obtained an accuracy of 0.706 using a fine-tuned CamemBERT prediction model with 2 classes, whereas we used 3 classes, which is more unfavorable. Kummervold et al [46] obtained an *F*_1_-score of 0.78 to predict the attitude of pregnant women toward vaccination against COVID-19, but the categories for the classification were different. Portelli et al [48] used a RoBERTa model trained on TweetEval Benchmark. However, the authors only provided the recall for sentiment analysis, which was 72.1, and not the precision, which does not allow a comparison with our results.

To improve our results, we propose using multitask training or pretrained embeddings. Multitask learning may improve results in some cases, but it is not systematic. This confirms that data science is an empirical discipline in which only experiments make it possible to determine the most appropriate approach in a defined context and that there is no method that makes it possible to confidently improve the results. In some cases, multitask learning made it possible to improve the results [45,62,63]. However, previous studies, such as that by Rodriguez et al [64], observed that multitask learning did not improve the results in general. However, it was possible to train the model on a simple task using transfer learning from the multitask model, obtaining better performance using less data. For the detection of fake news on COVID-19, Malla and Alphonse [65] showed how it could be interesting to use a CT-BERT (COVID-Twitter-BERT) model [25] but not a BERTweet model [66]. In 2020, Lee et al [67] proposed BioBERT, a language model pretrained on PubMed abstracts and PubMed Central full-text articles, that largely outperformed BERT for biomedical NLP tasks. Similarly, Med-BERT, a pretrained model on a structured electronic health record data set of 28,490,650 patients [68], allows working with smaller training sets while obtaining better performance.

In this study, we used Twitter rather than other social media platforms such as Facebook or Reddit. In fact, Wawrzuta et al [69] suggested that Facebook is more appropriate when evaluating public opinion because it has more active users compared with Twitter and Instagram and it is more representative of society’s demography than Twitter and Instagram, on which young city dwellers with a good level of education are overrepresented. However, Facebook does not provide an API allowing it to extract posts based on keywords. This is why it is necessary to clearly define and identify the discussion groups on which the study will focus [70]. Furthermore, it can be assumed that a poor selection of these groups could lead to biases when interpreting the data. One of the interests with Reddit, a discussion forum, is that it is divided into subreddits for which one can have an overview of the posts that relate to a place or a particular topic. This is not the case with Twitter on which the discussions concern rather a vast community and do not make it possible to know the specifics of the discussions in regional communities [71].

### Tweet Content Analysis

Throughout the health crisis, the media maintained a high level of attention on treatments against COVID-19. For example, Professor Didier Raoult, an internationally renowned French microbiologist [72], presented a study from the Institut Hospitalier Universitaire de Marseille, in which a treatment combining hydroxychloroquine and azithromycin made it possible to significantly decrease the viral load in patients with COVID-19 [73]. Part of the population was then doubtful of the reasons why health authorities did not authorize using these drugs. Believing that some therapeutic means are highly efficient at curing the disease makes it less desirable to apply preventive measures, which might have affected the perception of potential vaccines against COVID-19. The neighborhood of “hydroxychloroquine,” “vaccination,” and “Raoult” observed with the PCA in Figure 2 confirms our assumption that the perception of vaccines should be considered in relation to the perception of hydroxychloroquine. Additionally, a large portion of negative posts identified with our classifier mentioned chloroquine or hydroxychloroquine or tweets about Didier Raoult. However, PCA does not allow the evaluation of whether believing a treatment is efficient for quick healing may influence the intention of individuals to protect themselves, because the proximity relations on the PCA are not categorized. Nevertheless, further experiments with data from more recent time periods could help to determine if such relations should also be considered between vaccines and other presumed treatments such as ivermectin [74] or azithromycin [75]. As we found pharmaceutical companies that are only relevant to the vaccine or the COVID-19 crisis (eg, Gilead proposing an alternative treatment) and not others, we can assume that we applied a successful keyword selection strategy able to select relevant keywords. Our analysis revealed the dominant discourses and weak signals of vaccine mistrust.

After annotating a subset of tweets, an efficient classification implementing the state-of-the-art of NLP was able to reveal temporal trends in sentiment about potential vaccination against COVID-19. Moreover, further explorations of the vocabulary gave a different view of vaccine mistrust arguments.

Hence, there appears to be a change in the profile of Twitter users on this issue. According to Massey et al [76], Twitter seemed to be used more by profiles confident in the HPV vaccine, whereas our study showed a greater sharing of opposition against the announced COVID-19 vaccine, notably driven by political mistrust. Skepticism already observed with other vaccines could influence people who remain uncertain about vaccination because of the large audience that distrustful tweets have.

These observations may help the regulatory authorities to disseminate credible information by providing clear, precise communication around a potential campaign. The success of a vaccination campaign depends not only on sufficient coverage of the population to obtain collective immunity but also, and above all, on the acceptance of such a campaign by the same population. This study tends to clarify reasons for vaccine mistrust based on users’ reactions on social media.

### Limitations

The main limitation is that the annotation was performed by a single annotator. Additional experiments involving additional annotators have shown that annotations can be different in a very large number of tweets. This certainly reflects significant subjectivity related to the interpretation of the content of tweets in the chosen field. It would be interesting to take into account the role of emoticons in tweets because they can possibly help to better interpret the content, especially in the case of ironic content that we risk taking at face value.

This study focused on exploiting the textual information of Twitter but did not extract any further metadata such as users’ information. However, a preliminary experiment that we conducted earlier showed that medical professionals seem to be excluded from the debate on Twitter, except for a few personalities who are against a potential vaccine. This could lead to a better understanding of the observed dynamics.

Another limitation rests on the performances of the sentiment analysis. The model could achieve better performances in the near future with better parameter optimization and further exploration of other approaches. Models that are unsupervised like zero-shot learning could be interesting for additional investigation. Finally, it is important to emphasize that this study is not representative of neither the French population nor Twitter users in general.

### Conclusion

We proposed an approach that allows the selection of keywords using word embeddings with FastText and obtain a visual representation according to their closeness to one another with PCA. Then, we implemented a classifier using BERT that allows the prediction of the opinion on COVID-19 vaccines by Twitter users. Our study showed that Twitter could be a useful tool to investigate the arguments around vaccine mistrust (eg, the role of 5G in vaccination and discussions about other treatments such as chloroquine). Our results unveil that political aspects of vaccination overshadow its usual criticisms about adverse drug reactions. As the opposition rhetoric is generally more homogenous and more widely spread than the positive rhetoric, we believe that this study provides effective tools to help health authorities better understand vaccine mistrust.

## Appendix

Multimedia Appendix 1 List of 24 word pairs in French used for the Discounted Cumulative Gain (DCG).

Multimedia Appendix 2 List of the 69 selected keywords.

## Abbreviations

API: application programming interface
BERT: Bidirectional Encoder Representations from Transformers
DCG: Discounted Cumulative Gain
HPV: human papillomavirus
NLP: natural language processing
PCA: principal component analysis
TF-IDF: term frequency-inverse document frequency
VADER: Valence Aware Dictionary for Sentiment Reasoning
WHO: World Health Organization

## Data Availability

The original data set providing the tweets’ IDs can be found on the Panacea Lab GitHub site [77]. The code is available at [78], and the annotated training set is available on request.

## References

[1] MacDonald NE, SAGE Working Group on Vaccine Hesitancy (2015). Vaccine hesitancy: Definition, scope and determinants. Vaccine.

[2] Ward JK, Alleaume C, Peretti-Watel P, COCONEL Group (2020). The French public's attitudes to a future COVID-19 vaccine: The politicization of a public health issue. Soc Sci Med.

[3] Wakefield A, Murch S, Anthony A, Linnell J, Casson D, Malik M, Berelowitz M, Dhillon A, Thomson M, Harvey P, Valentine A, Davies S, Walker-Smith J (1998). Ileal-lymphoid-nodular hyperplasia, non-specific colitis, and pervasive developmental disorder in children. Lancet.

[4] Deer B (2011). How the case against the MMR vaccine was fixed. BMJ.

[5] Peretti-Watel P, Seror V, Cortaredona S, Launay O, Raude J, Verger P, Fressard L, Beck F, Legleye S, L'Haridon O, Léger D, Ward JK (2020). A future vaccination campaign against COVID-19 at risk of vaccine hesitancy and politicisation. The Lancet Infectious Diseases.

[6] Detoc M, Bruel S, Frappe P, Tardy B, Botelho-Nevers E, Gagneux-Brunon A (2020). Intention to participate in a COVID-19 vaccine clinical trial and to get vaccinated against COVID-19 in France during the pandemic. Vaccine.

[7] (2020). Global Attitudes on a COVID-19 Vaccine. IPSOS.

[8] Lazarus JV, Ratzan SC, Palayew A, Gostin LO, Larson HJ, Rabin K, Kimball S, El-Mohandes A (2021). A global survey of potential acceptance of a COVID-19 vaccine. Nat Med.

[9] Neumann-Böhme S, Varghese NE, Sabat I, Barros PP, Brouwer W, van Exel J, Schreyögg J, Stargardt T (2020). Once we have it, will we use it? A European survey on willingness to be vaccinated against COVID-19. Eur J Health Econ.

[10] Freeman D, Loe BS, Yu L, Freeman J, Chadwick A, Vaccari C, Shanyinde M, Harris V, Waite F, Rosebrock L, Petit A, Vanderslott S, Lewandowsky S, Larkin M, Innocenti S, Pollard AJ, McShane H, Lambe S (2021). Effects of different types of written vaccination information on COVID-19 vaccine hesitancy in the UK (OCEANS-III): a single-blind, parallel-group, randomised controlled trial. The Lancet Public Health.

[11] Wang X, Zou C, Xie Z, Li D Public Opinions towards COVID-19 in California and New York on Twitter. medRxiv. Preprint posted online July 14, 2020.

[12] Luo X, Zimet G, Shah S (2019). A natural language processing framework to analyse the opinions on HPV vaccination reflected in twitter over 10 years (2008 - 2017). Human Vaccines & Immunotherapeutics.

[13] Sha H, Al Hasan M, Mohler G, Brantingham PJ Dynamic topic modeling of the COVID-19 Twitter narrative among U.S. governors and cabinet executives. arXiv. Preprint posted online April 19, 2020.

[14] Daughton AR, Shelley CD, Barnard M, Gerts D, Watson Ross C, Crooker I, Nadiga G, Mukundan N, Vaquera Chavez NY, Parikh N, Pitts T, Fairchild G (2021). Mining and validating social media data for COVID-19-related human behaviors between January and July 2020: infodemiology study. J Med Internet Res.

[15] Chen S, Zhou L, Song Y, Xu Q, Wang P, Wang K, Ge Y, Janies D (2021). A novel machine learning framework for comparison of viral COVID-19-related Sina Weibo and Twitter posts: workflow development and content analysis. J Med Internet Res.

[16] Adikari A, Nawaratne R, De Silva D, Ranasinghe S, Alahakoon O, Alahakoon D (2021). Emotions of COVID-19: content analysis of self-reported information using artificial intelligence. J Med Internet Res.

[17] Ahmed N, Quinn SC, Hancock GR, Freimuth VS, Jamison A (2018). Social media use and influenza vaccine uptake among White and African American adults. Vaccine.

[18] Wawrzuta D, Jaworski M, Gotlib J, Panczyk M (2021). Characteristics of antivaccine messages on social media: systematic review. J Med Internet Res.

[19] Massey PM, Kearney MD, Hauer MK, Selvan P, Koku E, Leader AE (2020). Dimensions of misinformation about the HPV vaccine on Instagram: content and network analysis of social media characteristics. J Med Internet Res.

[20] Skeppstedt M, Kerren A, Stede M (2018). Vaccine Hesitancy in Discussion Forums: Computer-Assisted Argument Mining with Topic Models. The Communication Initiative Network.

[21] Zhang L, Fan H, Peng C, Rao G, Cong Q (2020). Sentiment analysis methods for HPV vaccines related tweets based on transfer learning. Healthcare (Basel).

[22] Hussain A, Tahir A, Hussain Z, Sheikh Z, Gogate M, Dashtipour K, Ali A, Sheikh A (2021). Artificial intelligence-enabled analysis of public attitudes on Facebook and Twitter toward COVID-19 vaccines in the United Kingdom and the United States: observational study. J Med Internet Res.

[23] Kwok SWH, Vadde SK, Wang G (2021). Tweet topics and sentiments relating to COVID-19 vaccination among Australian Twitter users: machine learning analysis. J Med Internet Res.

[24] Blei DM, Ng AY, Jordan MI (2003). Latent Dirichlet allocation. Journal of Machine Learning Research.

[25] Müller M, Salathé M, Kummervold PE COVID-Twitter-BERT: a natural language processing model to analyse COVID-19 content on Twitter. arXiv. Preprint posted online May 15, 2020.

[26] Arnaud M, Elbattah M, Gignon M, Dequen G (2022). Learning embeddings from free-text triage notes using pretrained transformer models. HEALTHINF: Proceedings of the 15th International Joint Conference on Biomedical Engineering Systems and Technologies.

[27] Blanc C, Bailly A, Guillotin T, Jamal F, Wakim B, Roy P, Francis (2022). FlauBERT vs. CamemBERT: Understanding patient's answers by a French medical chatbot. Artif Intell Med.

[28] Sauvayre R, Vernier J, Chauvière C (2022). An analysis of French-language tweets about COVID-19 vaccines: supervised learning approach. JMIR Med Inform.

[29] Ten threats to global health in 2019. World Health Organization.

[30] Taylor S, Landry CA, Paluszek MM, Groenewoud R, Rachor GS, Asmundson GJG (2020). A proactive approach for managing COVID-19: the importance of understanding the motivational roots of vaccination hesitancy for SARS-CoV2. Front Psychol.

[31] Jolley D, Douglas KM (2014). The effects of anti-vaccine conspiracy theories on vaccination intentions. PLoS One.

[32] Shahsavari S, Holur P, Wang T, Tangherlini TR, Roychowdhury V (2020). Conspiracy in the time of corona: automatic detection of emerging COVID-19 conspiracy theories in social media and the news. J Comput Soc Sci.

[33] Audureau W (2020). Anti-vaccine discourse, well established in France, has redoubled its vigor with the health crisis. Le Monde.

[34] Mehra MR, Desai SS, Ruschitzka F, Patel AN (2020). RETRACTED: Hydroxychloroquine or chloroquine with or without a macrolide for treatment of COVID-19: a multinational registry analysis. The Lancet.

[35] Mehra MR, Desai SS, Kuy S, Henry TD, Patel AN (2020). Retraction: cardiovascular disease, drug therapy, and mortality in Covid-19. N Engl J Med.

[36] Vosoughi S, Roy D, Aral S (2018). The spread of true and false news online. Science.

[37] The Lancet Infectious Diseases (2020). The COVID-19 infodemic. The Lancet Infectious Diseases.

[38] Burki T (2020). The online anti-vaccine movement in the age of COVID-19. The Lancet Digital Health.

[39] Grimes DR (2020). Health disinformation & social media: The crucial role of information hygiene in mitigating conspiracy theory and infodemics. EMBO Rep.

[40] Banda JM, Tekumalla R, Wang G, Yu J, Liu T, Ding Y, Artemova E, Tutubalina E, Chowell G (2021). A large-scale COVID-19 Twitter chatter dataset for open scientific research-an international collaboration. Epidemiologia (Basel).

[41] Devlin J, Chang MW, Lee K, Toutanova K (2019). BERT: pre-training of deep Bidirectional Transformers for language understanding. Proceedings of the 2019 Conference of the North American Chapter of the Association for Computational Linguistics: Human Language Technologies, Volume 1 (Long and Short Papers).

[42] Martin L, Muller B, Suárez PJO, Dupont Y, Romary L, de la Clergerie EV, Seddah D, Sagot B CamemBERT: a tasty French language model. arXiv. Preprint posted online May 21, 2020.

[43] Hutto C, Gilbert E (2014). VADER: a parsimonious rule-based model for sentiment analysis of social media text. Proceedings of the International AAAI Conference on Web and Social Media.

[44] Visweswaran S, Colditz JB, O'Halloran P, Han N, Taneja SB, Welling J, Chu K, Sidani JE, Primack BA (2020). Machine learning classifiers for Twitter surveillance of vaping: comparative machine learning study. J Med Internet Res.

[45] Hussain S, Afzal H, Saeed R, Iltaf N, Umair MY (2021). Pharmacovigilance with transformers: a framework to detect adverse drug reactions using BERT fine-tuned with FARM. Comput Math Methods Med.

[46] Kummervold PE, Martin S, Dada S, Kilich E, Denny C, Paterson P, Larson HJ (2021). Categorizing vaccine confidence with a transformer-based machine learning model: analysis of nuances of vaccine sentiment in Twitter discourse. JMIR Med Inform.

[47] Monselise M, Chang C, Ferreira G, Yang R, Yang CC (2021). Topics and sentiments of public concerns regarding COVID-19 vaccines: social media trend analysis. J Med Internet Res.

[48] Portelli B, Scaboro S, Tonino R, Chersoni E, Santus E, Serra G (2022). Monitoring user opinions and side effects on COVID-19 vaccines in the Twittersphere: infodemiology study of tweets. J Med Internet Res.

[49] Mønsted B, Lehmann S (2022). Characterizing polarization in online vaccine discourse-A large-scale study. PLoS One.

[50] Bokaee Nezhad Z, Deihimi MA (2022). Twitter sentiment analysis from Iran about COVID 19 vaccine. Diabetes Metab Syndr.

[51] Rahmanti AR, Chien C, Nursetyo AA, Husnayain A, Wiratama BS, Fuad A, Yang H, Li YJ (2022). Social media sentiment analysis to monitor the performance of vaccination coverage during the early phase of the national COVID-19 vaccine rollout. Comput Methods Programs Biomed.

[52] Chinnasamy P, Suresh V, Ramprathap K, Jebamani BJA, Srinivas Rao K, Shiva Kranthi M (2022). COVID-19 vaccine sentiment analysis using public opinions on Twitter. Mater Today Proc.

[53] Bari A, Heymann M, Cohen RJ, Zhao R, Szabo L, Apas Vasandani S, Khubchandani A, DiLorenzo M, Coffee M (2022). Exploring coronavirus disease 2019 vaccine hesitancy on Twitter using sentiment analysis and natural language processing algorithms. Clin Infect Dis.

[54] Chandrasekaran R, Desai R, Shah H, Kumar V, Moustakas E (2022). Examining public sentiments and attitudes toward COVID-19 vaccination: infoveillance study using Twitter posts. JMIR Infodemiology.

[55] Li L, Zhou J, Ma Z, Bensi MT, Hall MA, Baecher GB (2022). Dynamic assessment of the COVID-19 vaccine acceptance leveraging social media data. J Biomed Inform.

[56] Roe C, Lowe M, Williams B, Miller C (2021). Public perception of SARS-CoV-2 vaccinations on social media: questionnaire and sentiment analysis. Int J Environ Res Public Health.

[57] Huangfu L, Mo Y, Zhang P, Zeng DD, He S (2022). COVID-19 vaccine tweets after vaccine rollout: sentiment-based topic modeling. J Med Internet Res.

[58] Karami A, Zhu M, Goldschmidt B, Boyajieff HR, Najafabadi MM (2021). COVID-19 vaccine and social media in the U.S.: exploring emotions and discussions on Twitter. Vaccines (Basel).

[59] Liew TM, Lee CS (2021). Examining the utility of social media in COVID-19 vaccination: unsupervised learning of 672,133 Twitter posts. JMIR Public Health Surveill.

[60] Yousefinaghani S, Dara R, Mubareka S, Papadopoulos A, Sharif S (2021). An analysis of COVID-19 vaccine sentiments and opinions on Twitter. Int J Infect Dis.

[61] He L, Yin T, Zheng K (2022). They May Not Work! An evaluation of eleven sentiment analysis tools on seven social media datasets. J Biomed Inform.

[62] Kades K, Sellner J, Koehler G, Full PM, Lai TYE, Kleesiek J, Maier-Hein KH (2021). Adapting Bidirectional Encoder Representations from Transformers (BERT) to assess clinical semantic textual similarity: algorithm development and validation study. JMIR Med Inform.

[63] Zhu Y, Liang X, Batsis JA, Roth RM (2021). Exploring deep transfer learning techniques for Alzheimer's dementia detection. Front Comput Sci.

[64] Rodriguez NE, Nguyen M, McInnes BT (2022). Effects of data and entity ablation on multitask learning models for biomedical entity recognition. J Biomed Inform.

[65] Malla S, Alphonse PJA (2022). Fake or real news about COVID-19? Pretrained transformer model to detect potential misleading news. Eur Phys J Spec Top.

[66] Nguyen DQ, Vu T, Nguyen AT (2020). BERTweet: A pre-trained language model for English tweets. Proceedings of the 2020 Conference on Empirical Methods in Natural Language Processing: System Demonstrations.

[67] Lee J, Yoon W, Kim S, Kim D, Kim S, So CH, Kang J (2020). BioBERT: a pre-trained biomedical language representation model for biomedical text mining. Bioinformatics.

[68] Rasmy L, Xiang Y, Xie Z, Tao C, Zhi D (2021). Med-BERT: pretrained contextualized embeddings on large-scale structured electronic health records for disease prediction. NPJ Digit Med.

[69] Wawrzuta D, Jaworski M, Gotlib J, Panczyk M (2021). What arguments against COVID-19 vaccines run on Facebook in Poland: content analysis of comments. Vaccines (Basel).

[70] Kalichman SC, Eaton LA, Earnshaw VA, Brousseau N (2022). Faster than warp speed: early attention to COVD-19 by anti-vaccine groups on Facebook. J Public Health (Oxf).

[71] Yan C, Law M, Nguyen S, Cheung J, Kong J (2021). Comparing public sentiment toward COVID-19 vaccines across Canadian cities: analysis of comments on Reddit. J Med Internet Res.

[72] Raoult D (2020). Coronavirus: Towards a Way Out of the Crisis?. Mediterranean Infection.

[73] Gautret P, Lagier JC, Parola P, Hoang VT, Meddeb L, Mailhe M, Doudier B, Courjon J, Giordanengo V, Vieira VE, Tissot Dupont H, Honoré S, Colson P, Chabrière E, La Scola B, Rolain JM, Brouqui P, Raoult D (2020). Hydroxychloroquine and azithromycin as a treatment of COVID-19: results of an open-label non-randomized clinical trial. Int J Antimicrob Agents.

[74] López-Medina E, López P, Hurtado IC, Dávalos DM, Ramirez O, Martínez E, Díazgranados JA, Oñate JM, Chavarriaga H, Herrera S, Parra B, Libreros G, Jaramillo R, Avendaño AC, Toro DF, Torres M, Lesmes MC, Rios CA, Caicedo I (2021). Effect of ivermectin on time to resolution of symptoms among adults with mild COVID-19: a randomized clinical trial. JAMA.

[75] Oldenburg CE, Pinsky BA, Brogdon J, Chen C, Ruder K, Zhong L, Nyatigo F, Cook CA, Hinterwirth A, Lebas E, Redd T, Porco TC, Lietman TM, Arnold BF, Doan T (2021). Effect of oral azithromycin vs placebo on COVID-19 symptoms in outpatients with SARS-CoV-2 infection: a randomized clinical trial. JAMA.

[76] Massey PM, Leader A, Yom-Tov E, Budenz A, Fisher K, Klassen AC (2016). Applying multiple data collection tools to quantify human papillomavirus vaccine communication on Twitter. J Med Internet Res.

[77] thepanacealab / covid19_twitter. GitHub.

[78] ChristelDG / Covid_Vax_Tweet_Classif. GitHub.

