# Supplementary material for: Users’ Reactions to Announced Vaccines Against COVID-19 Before Marketing in France: Analysis of Twitter Posts
Source: J Med Internet Res. 2023 Apr 24;25:e37237. doi: 10.2196/37237 (PMC10132828; doi:10.2196/37237)
Supplement: Multimedia Appendix 1 [file jmir_v25i1e37237_app1.docx]

| ["covid","coronavirus"] | ["covid", "coronavirus"] |
| --- | --- |
| ["epidemie","pandemie"] | ["epidemic", "pandemic"] |
| ["president","republique"] | ["president","republic"] |
| ["crise","sanitaire"] | ["crisis", "sanitary"] |
| ["traitement","vaccin"] | ["treatment","vaccine"] |
| ["soignants","infirmiers"] | ["caregivers", "nurses"] |
| ["deces","morts"] | ["death","dead"] |
| ["mai","avril"] | ["May","April"] |
| ["vague","seconde"] | ["wave", "second"] |
| ["chloroquine","raoult"] | ["chloroquine","raoult"] |
| ["cas","confirmes"] | ["cases","confirmed"] |
| ["gels","masques"] | ["gels","masks"] |
| ["gestes","barrieres"] | ["gestures", "barriers"] |
| ["positifs","detectes"] | ["positive", "detected"] |
| ["frontieres","touristes"] | ["borders", "tourists"] |
| ["immunite","collective"] | ["immunity","herd"] |
| ["quarantaine","isolement"] | ["quarantine", "isolation"] |
| ["penurie","manque"] | ["shortage", "lack"] |
| ["conseil","scientifique"] | ["scientific", "Council"] |
| ["bill","gates"] | ["bill","gates"] |
| ["chomage","partiel"] | ["Partial", "unemployment"] |
| ["urgence","sanitaire"] | ["health", "emergency"] |
| ["attestation","deplacement"] | ["certificate", "travel"] |
| ["departement","region"] | ["department","region"] |
