# Supplementary material for: Users’ Reactions to Announced Vaccines Against COVID-19 Before Marketing in France: Analysis of Twitter Posts
Source: J Med Internet Res. 2023 Apr 24;25:e37237. doi: 10.2196/37237 (PMC10132828; doi:10.2196/37237)
Supplement: Multimedia Appendix 2 [file jmir_v25i1e37237_app2.docx]

| Antibiotique | Antibiotic |
| --- | --- |
| Antidote | Antidote |
| Antiviral | Antiviral |
| Antiviraux | Antivirals |
| Artemisia | artemisia |
| Astrazeneca | Astrazeneca |
| Automédication | Self-medication |
| Azithromycin | Azithromycin |
| Azythromycin | Azythromycin |
| Bigpharma | bigpharma |
| Blackrock | Blackrock |
| Charlatan | Charlatan |
| Chloroquine | Chloroquine |
| Cobaye | Guinea pig |
| Competent | Competent |
| Complot | Conspiracy |
| Conflit | Conflict |
| Conspiration | Conspiracy |
| Corrompu | Corrupt |
| Dexamethasone | Dexamethasone |
| Didier | Didier |
| Dose | Dose |
| Escroc | Crook |
| Essais | Trials |
| Etude | Study |
| Gates | Gates |
| Gilead | Gilead |
| Gsk | gsk |
| Hydroxychloroquine | Hydroxychloroquine |
| Inject | Inject |
| Intérêt | Interest |
| Labos | Labs |
| Lancet | Lancet |
| Lobbies | Lobbies |
| Médicament | Medication |
| Medoc | Slang term for “drug” |
| Miracle | Miracle |
| Moderna | Moderna |
| Mondialiste | globalist |
| Novartis | Novartis |
| Oxford | Oxford |
| Pfizer | Pfizer |
| Pharmaceutique | Pharmaceutical |
| Plaquenil | Plaquenil |
| Potion | Potion |
| Prophylaxie | Prophylaxis |
| Puce | Chip |
| Raoult | Raoult |
| Remdesevir | Remdesevir |
| Remède | Remedy |
| Rfid | RFID |
| Sanofi | Sanofi |
| Soigner | Cure |
| Soros | Soros |
| Stéroide | Steroid |
| Surgisphere | Surgisphere |
| Theorie | Theory |
| Tisane | Tisane |
| Tocilizumab | Tocilizumab |
| Toxi | Toxic |
| Traitement | Treatment |
| Traitre | Traitor |
| Vaccin | Vaccine |
| Vaccination | Vaccination |
| Vacciner | Vaccinate |
| Virologue | Virologist |
